# Bad to All? A Novel Way to Analyze the Effects of Fee-for-Service on Multiple Grades Hospitals Operation Outcomes

**DOI:** 10.3390/ijerph182312723

**Published:** 2021-12-02

**Authors:** Yiting Wang, Wenhui Hou, Xiaokang Wang, Hongyu Zhang, Jianqiang Wang

**Affiliations:** 1 School of Business, Central South University, Changsha 410083, China; 181601043@csu.edu.cn (Y.W.); wenhuih@csu.edu.cn (W.H.); xkwang@csu.edu.cn (X.W.); hyzhang@csu.edu.cn (H.Z.); 2 School of Finance, Hunan University of Finance and Economics, Changsha 410205, China; 3 Hunan Engineering Research Center for Intelligent Decision Making and Big Data on Industrial Development, Hunan University of Science and Technology, Xiangtan 411201, China

**Keywords:** Fee-for-Service, hospital operation outcomes, classification of hospitals, reimbursement scheme

## Abstract

It is a consensus that Fee-for-Service (FFS) is a traditional medical insurance payment scheme with significant disadvantages, namely the waste of health care resources. However, the majority of the prior works that draw such conclusions from the perspective of social welfare while analyzing the impacts of FFS on operation outcomes of hospitals still lack attention from the existing literature, considering the fact that the majority of public hospitals are self-founding. Under this motivation, we collected operation data of 301 public hospitals with different grades (grade II and III) in central China. Here, we present a novel statistical evaluation framework on the impact of FFS on hospital operation outcomes from four dimensions (financial income, efficiency, medical service capacity, and sustainability) using fixed-effects multivariate regression. With verification by the robustness test, our results indicate that: (i) The classification of the hospital (COH) significantly affected the impacts of FFS on hospitals’ operations. (ii) For grade III hospitals, FFS leads to higher financial income, medical service capacity (MSC) and longer length-of-stay (LOS). (iii) However, as for grade II hospitals, hospitals with FFS adoptions achieve lower financial income, lower MSC and shorter LOS, which violates the common sense from previous works. (iv) FFS has a significant negative impact on public hospital’s sustainable development; however, there is lack of evidence showing that sustainability would be affected by the interaction effects between FFS and COH. We believe these new findings from the perspective of hospital operation provide insights and could serve as a reference for the healthcare payment hierarchical reform by COH in low and middle-income countries (LMICs), which are going through the primary stage of the healthcare reform.

## 1. Introduction

In the medical market, the provider payment method is a fundamental approach to organize healthcare resources and guide the behaviors of healthcare providers [1].

Nowadays, the post-payment system, Fee-for-Service (FFS), is still the most prevailing payment method of health providers in low and middle-income countries (LMICs) [2], e.g., central Asia and Eastern Europe [3,4]. Under FFS, medical fees are charged based on medical services prescribed by doctors, such as examinations, drugs, and surgeries, which are simple to administer. However, prior works [5,6] reported that healthcare providers are financially incentivized to prescribe more expensive and profitable medications or diagnostic tests, which may not always be necessary to patients under FFS scheme [7]. This leads to increased health care costs and waste of public healthcare resources. Thus, it is a consensus that FFS is responsible mainly for excessive health care services, inefficiencies, uncontrolled health costs, and even violation of medical ethics [5,7,8,9,10,11].

To contain costs and effectiveness of the healthcare system, many developed countries have opted to reform the payment system. For example, the United States Medicare program [12] and Japan payment model shift [8] replaced traditional FFS with a prospective payment system (PPS) in 1983 and 2003 respectively. Being motivated by these successful examples, many LMICs launch their medical reform with payment scheme shift by abandoning FFS scheme [2].

However, we indicate that impact of FFS on the medical service system is not yet be thoroughly studied:

- Most of the existing works criticize FFS leads to over-treatment from the perspective of social welfare. Nonetheless, the majority of public hospitals are self-founding. Evaluating the impacts of FFS on hospitals operation outcomes from multiple dimensions as business entities is still absent from the existing literature.
- Classification of Hospitals (COH) has played an active role in establishing an efficient health service system by regulating different grade hospitals to provide multiple levels and discrepant items of service to the patients [13]. Thus, the payment scheme could have a distinct impact on a different classes of hospitals. Unfortunately, the impact of FFS on different grades of hospitals has not been addressed in the prior works.

Therefore, if FFS does no benefits only harm from social welfare and hospital operation perspectives as common sense in previous works? If FFS has the same impacts on the operation of hospitals with different COH?

To answer these two opening questions, we empirically analyze the effectiveness of the FFS payment scheme on hospital operation outcomes such as financial income, efficiency, medical service capacity, and sustainability. Besides, we also investigate the influence of COH on FFS’s impactions on hospital operations by checking whether FFS and COH have an interaction effect.

### 1.1. Background and Related Works

#### 1.1.1. Background

##### Classification of Hospitals in China

Different COH plays a vital role in health care in China. According to their functions and roles, the public hospitals are divided into grade I, II, and III. The criteria of COH rating include the size, the technical level, the medical equipment, the management level, and the quality of medical service. COH mainly represents the hospital’s size, which is reflected by the number of hospital beds, building area, department settings, staffing, and the other hardware facilities standards. The most immediate effect of COH is the pricing for various medical services, namely, the higher level the hospital is, the higher pricing of medical services is [14]. Besides, it also affects the government subsidies and hospitals’ reputations at the same time. Taken together, the higher-grade hospitals with a larger corresponding construction scale, more detailed department settings, more advanced equipment, and more mature technical levels can provide higher overall quality of medical services, thereby attracting more patients [15].

Grade I hospitals are primary health care institutions that directly provide prevention, medical care, and rehabilitation services to residents with common diseases and make correct referrals for serious and difficult diseases. Grade II hospitals are regional hospitals that provide comprehensive medical services across several communities, receive referrals from Grade I hospitals, and undertake certain scientific research and teaching tasks. Grade III hospitals are tertiary hospitals that provide high-level specialized medical services to solve critical and difficult medical conditions, receive referrals from grade II hospitals, and undertake advanced scientific research and teaching tasks.

COH has played an active role in establishing an efficient health care administration system, strengthening the three-tier prevention healthcare network, and providing accessible and appropriate medical services [16]. However, the uneven distribution of medical resources under COH is becoming a severe problem in China, which is reflected in the contradiction between people’s demand for high-quality medical services and the unbalanced allocation and inadequate medical resources [17].

#### 1.1.2. Related Works

In this section, we first review the existing literature related to the effectiveness of medical payment schemes from different perspectives.

##### Analysis FFS Scheme from Social Welfare Perspective

In the existing works, the effectiveness of FFS on multiple social welfare aspects has been extensively studied, such as the overprovision problem, patients behavior, and medical ethics problem caused by FFS. Di Guida et al. [18] tested the extent of overprovision under FFS. They concluded that the risk of overprovision under FFS depends on fee sizes, patients’ health profiles, and market conditions. In contrast, Wagstaff and Lindelow [19] investigated the FFS effects from patients’ behavior and indicated that FFS increases the risk of high and catastrophic health care spending. Further analysis suggests that this is due to FFS encouraging patients to seek care when sick and to seek care from higher-level providers. Besides, Yip et al. [20] addressed the erosion of medical ethics problem arisen by the FFS scheme in China.

Although FFS has been evaluated from different social welfare dimensions, the evaluation on the impacts of FFS on hospitals operation outcomes is lack of attention from the existing literatures.

##### Analysis Non-FFS Schemes from Hospital Operation Perspective

Sort of the effectiveness of new payment schemes such as bundle payment (BP), value-based purchasing (VBP), clinical practice variation, and health information technology (HIT) on hospital operation has been studied.

Meng et al. [21] conducted a Difference-in-Differences (DID) analysis to analyze the implementation of episode-based BP (EBP) policy on childbirth and empirically found that EBP led to a reduction of length-of-stay (LOS). While whether and how the VBP penalties affect aggregate operating outcomes of healthcare providers in hospitals are reported in [22]. Besides, some interesting factors are being evaluated. For example, Ref. [23] explored whether and how lower variations in clinical practice relate to hospital operation. In addition, Sharma et al. [24] examined how two essential HIT bundles jointly impact cost and process quality outcomes.

Even though the effectiveness of multiple new payment schemes on hospital operation has been addressed before, surprisingly, the effectiveness of traditional FFS scheme on the hospital operation outcomes have rarely been emprically analyzed.

##### Analysis FFS Scheme on Hospital Performance

Refs. [8,25], which focus on evaluating the impacts of FFS on hospital performance, are two works most related to this paper.

Adida et al. [8] used a model-based approach for comparing the performance of FFS and BP for healthcare services. FFS provides incentives for excessive treatment intensity and results in suboptimal system payoff. At the same time, BP could lead to suboptimal patient selection and treatment levels that may be lower or higher than desirable for the system, with a high level of financial risk for the provider. They also found that the performance of BP is extremely sensitive to the bundled payment value and to the provider’s risk aversion. Guo et al. [25] examined the impact of FFS and BP reimbursement schemes on the patient revisit rate. The focus of the analysis is on FFS and BP. They found that when the patient pool is large, the BP scheme dominates in terms of lower revisit rate than FFS.

Note that both [8,25] use a single variable to measure the hospital operation performance; namely financial income and patient revisit rate, by using the data without distinguishing COH.

In summary, by reviewing the related works above, we want to highlight that investigation of the effectiveness of FFS on hospital operation from multiple dimensions by considering COH is absent from the existing works.

### 1.2. Motivations

In this section, we briefly discuss the motivations and significance of this paper.

Why is it important to evaluate the effects on hospital operation outcomes?

According to China’s health statistical yearbook, in the past decade, the debt scale of public hospitals has increased sharply. In 2014, the total debt exceeded 1 trillion yuan, with an average annual compound growth rate of 20.5%. Moreover, the asset-liability ratio of public hospitals also increased from 27.12% in 2005 to 40.80% in 2014. There is a severe mismatch between the large-scale rapid expansion of public hospitals and the quality and effectiveness of hospital services, which force the public hospital to keep increasing the operational performance to maintain service capacity and quality [26,27].

Thus, analysis of the impacts of FFS, which is the most common payment in LMICs, on hospital operation outcomes will provide direct evidence to guide the reform of the medical payment system.

If FFS bad to both social welfare and hospital outcomes?

As we mentioned in the Section 1.1.2, there is a gap between current evidence of the effects of FFS payment on the social welfare system in the previous works and what is needed to inform health policymaking. Besides, the connections between FFS and COH on the hospital operation have not been revealed yet. Thus, it is not rigorous to conclude that the FFS scheme is an evil practice that must be stamped out without thoroughly studying its impacts from different perspectives and considering COH.

Therefore, one of the primary purposes of this paper is to investigate and give evidence of the effects of FFS on hospital operation outcomes together with the consideration of COH.

If FFS bad to all COH?

In most countries, hospitals will be classified into multiple grades that recognize a hospital’s ability to provide medical service, medical education, and conduct medical research such as community, regional and tertiary referral hospitals in the West, and grade I, II III hospitals in China. COH plays an essential role in establishing an efficient and effective healthcare service system by regulating different grade hospitals to provide multiple levels and discrepant items of service to the patients [13]. Thus, the payment scheme could have distinct impacts on different classifications of hospitals. Surprisingly, as discussed in Section 1.1.2, prior work can regard all the COH as a unified type of hospital [8] or as studies only a specific type of the hospital [28].

In summary, answers to the above three questions are essential but have not been thoroughly studied. Specifically, it is crucial to fill the gap between evidence of the effects of FFS payment on operation outcomes of different COH and what is needed to inform health policymaking. However, unfortunately, the effectiveness of FFS on hospital operation outcomes and the connections between FFS and COH are absent from the existing literature. Therefore, we present a new framework to examine the effectiveness of the reimbursement scheme FFS on public hospitals operations and whether FFS and COH have a cross effect on the operation of hospitals.

Who can benefit from this work?

By analyzing the effectiveness of the prevailing reimbursement scheme FFS on public hospitals’ operations with COH, and providing reasonable interpretations, hospital administrators, government policymakers, and the researchers within related topics will benefit from this work. (i) Firstly, hospital administrators can find deficiencies in the existing hospital management and the direction of future improvement from our research results, such as sustainability of long-term development. (ii) In addition, our research can also shed light on health care policy makers. Specifically, future medical payment reform may consider the systematic variation between grade II and grade III hospitals, and reflect those differences in policies, thus guiding the reform of both hospitals in the same direction. (iii) Besides, the researchers from related communities could analyze medical insurance payment on hospital performance based on our analytical framework to explore the impacts of other payment methods on hospital’s operation, such as DRG, DIP, Capitation, and so on. On the other hand, future research can also expand the dimensions based on our framework to analyze more operation management problems in health care and hospital research fields.

### 1.3. Contributions and Paper Organization

In this paper, we collected operation data of 301 public hospitals with different grades (grade II and III) in central China. Then we presented a novel statistical evaluation framework to the impact of FFS on hospital operation outcomes from four dimensions: financial income, efficiency, medical service capacity, and sustainability by using fixed-effects multivariate regression. Our results indicate that: (i) The classification of the hospital (COH) significantly affected the impacts of FFS on hospitals’ operations. (ii) For grade III hospitals, FFS leads to higher financial income, medical service capacity (MSC), and longer length-of-stay (LOS), which is similar to the prior works. (iii) However, regarding grade II hospitals, they achieve lower financial income, lower MSC, and shorter LOS, which violates the common sense in previous works. (iv) There is lack of evidence showing that sustainability would be affected by the interaction effects between FFS and COH.These new findings from the perspective of hospital operations provide insight and could serve as a reference for the healthcare payment hierarchical reform by COH in low and middle-income countries (LMICs), who are going through the primary stage of the reform of health care.

Our main contributions can be summarized as follows:

- We evaluate the effects of FFS from a new perspective, namely hospital operation outcomes, and leads to some counter-intuitive conclusions that FFS is not an all-disadvantages scheme when considering COH.
- To the best of our knowledge, this is the first work to empirically evaluate the effects of FFS on hospital outcomes from multiple dimensions over the single measurements in the previous works. Furthermore, this is also the first work to investigate the FFS impactions on different grades of hospitals operations. These new findings could provide insight and information to healthcare policymaking in the future.
- We present a novel statistical evaluation approach to the effects of reimbursement scheme on the hospital performance. We believe this can serve as a basic analysis framework to evaluate other reimbursement schemes and more complex measurements variables in other countries. We will publish the analysis source code and sample data via a source code link (https://github.com/YiTingWangCS/EffectHospital, accessed on 1 September 2021).

We organize the paper as follows: We first introduce the data collection and measurements in Section 2.1. We then present the analysis strategy on the effects of FFS on hospitals operation in Section 2.2. followed by presenting the results in Section 3. In Section 4, we provide an interpretation and discussion of the results. The conclusions are presented in Section 5.

## 2. Methodology

### 2.1. Data and Measurement

Data Source

We comprehensively consider the long-term development of the public hospitals from the following perspectives: financial income, efficiency, medical service capacity, and sustainability. We collected our needed data from the department of health commission and the d department of health care security administration, which covering 64 grade III public hospitals and 237 grade II public hospitals in a province in central China from 2016 to 2019. The collected hospitals’ structural characteristics are shown in Table 1.

We propose to use 1 independent variable, 4 dependent variables and 8 control variables to perform the statistical analysis. Table 2 summarizes and illustrates the variables used in this study.

Dependent variables

We used public grade II and grade III hospitals operation data extracted by National Health Commission (NHC) to assess the effectiveness of FFS on hospitals operations in four dimensions by using four proxy outcome measures, namely, annual medical revenue, annual average length-of-stay (LOS), annual total number of medical services provided and the annual amount of investment in medical personnel training:

- Financial income: Even though the public institutions are supposed to provide medical services for public benefit, and more than half of the hospital revenue is obtained from the healthcare security administration, the public hospital should be responsible for its own profits and losses. Thus, the survival of public hospitals is the basic guideline for hospital operation, and in this research, the medical revenue is used to reflect the financial situation of hospitals [29].
- Efficiency: LOS, a proxy measure for hospital resource usage, is widely used in hospital’s operations studies [23,29,30,31,32]. However, some scholars argue that using the length of stay for an individual discharge may be misleading since the readmissions could consume additional resources during an entire episode [33]. Thus, we measure the average LOS at the hospital level to reduce potential estimation bias due to the variation in stay lengths for operational performance [34].
- Medical service capacity: Annual total number of medical services provided is calculated by the sum of number of outpatients and number of discharged patients, which measures the capacity of hospitals to meet basic medical needs.
- Sustainability: The construction of building talent team and teaching and research ability, which can reflect the hospital’s sustainable development ability, is an important indicator reflecting the innovative development and sustainable and healthy operation of public hospitals, especially for public tertiary hospitals in China, for which provide high-level specialized medical services and undertake advanced teaching and scientific research tasks [16]. Thus, talents training is an important guarantee for healthy and sustainable development of medical and health institutions in China, and the importance attached to academic clinical training could realize the sustainable development and improve the overall performance of hospitals. Amount of investment in medical personnel training is one of the most intuitive input variable for evaluating hospital’s sustainability.

We take the natural logarithm of all the dependent variables to account for the heavy tails.

Control variables

Hospital operations are sophisticated for which it is affected by many aspects based on prior studies. we control for several variables that can cause variations in hospitals operating outcomes as well as factors that can vary systematically over time. We used a fixed effects estimator to identify the causal effects of FFS on the outcomes, and hence we control for the time variant characteristics to remove potential trend effects:

- Hospital level:Since the data we collected contains the information of hospital level, we used a binary variable to indicate if a hospital belongs to grade III (tertiary hospital) or not.
- Hospital size and Staff number: Hospital size by controlling bed size and the number of full-time employees [16,22,23,29,34,35]. We take the natural logarithm of beds and the number of employees to account for the heavy tails in this distribution.
- Total expenditure: A greater amount of expenditure for patient treatment may be caused by any chance of uncertain outcomes, whether it is due to the meaningful efforts of health care professionals in delivering appropriate care, or the wasteful practice when a patient receives excessive care [23]. We take the natural logarithm of this variable to eliminate the differences in performance outcomes caused by different hospital total expenditures.
- Ratio of incomes from health insurance funds: It has been reported that revenue of public hospitals from various insurance funds reached 51.5% of the income in public hospitals. The ratio of insurance funds becomes an important variable to measure the hospitals’ operation. Thus, we used the ratio of medical income derived from health insurance funds and the ratio of hospitalization income derived from health insurance funds as two control variables.
- Regional development level: Since the one of the main financial funding source of public hospital is from local finance support, thus, the regional development level directly affects the public hospitals’ operation. As a result, we use regional GDP per capita and the local total expenditure in healthcare as two control variables.

### 2.2. Analysis Strategy

Model selection

We ran the Hausman test (*p* < 0.05), and the results showed that we need to use a fixed-effect multivariate regression model to conduct our analysis.

Statistical analysis

We employ a Fixed Effects (FE) model to account for unobserved hospital specific effects, and the Hausman test further indicated the FE model is more appropriate (*p* < 0.05) for consistent estimates. The general regression model is as follows:

(1) $$\begin{array}{cc} DV_{jit}= & \beta_{0}+\beta_{1}{\mathrm{FFS}}_{it}+\beta_{2}{\mathrm{IsGrade}}_{it}+\beta_{3}{\mathrm{lnBed}}_{it}+\beta_{4}{\mathrm{lnNFE}}_{it}+\beta_{5}{\mathrm{lnTExpend}}_{it}\\ \; & +\beta_{6}{\mathrm{MediRatio}}_{it}+\beta_{7}{\mathrm{HospRatio}}_{it}+\beta_{8}{\mathrm{IPGDP}}_{it}+\beta_{9}{\mathrm{lnLPEHealth}}_{it}\\ \; & +{\mathrm{YearDummies}}_{t}+E_{i}+e_{it} \end{array}$$

where $DV_{jit}$ is our key dependent variables representing four outcomes of hospitals operations: financial income, LOS, medical service capacity and sustainability. $\beta_{0}$ is a constant number while $\beta_{1}$ is coefficient of independent variable FFS, $\beta_{2}$ to $\beta_{9}$ are coefficients of control variables. ${\mathrm{YearDummies}}_{t}$ is also a control variable. $E_{i}$ and $e_{it}$ represent time-invariant and idiosyncratic errors, respectively.

In order to examine whether FFS and COH have a cross effect on operation of hospitals. the regression model is set as follows:

(2) $$\begin{array}{cc} DV_{jit}= & \beta_{0}+\beta_{1}{\mathrm{FFS}}_{it}+\beta_{2}{\mathrm{IsGrade}}_{it}+\beta_{3}{\mathrm{FFS}}_{it}{\mathrm{IsGrade}}_{it}+\beta_{4}{\mathrm{lnBed}}_{it}+\beta_{5}{\mathrm{lnNFE}}_{it}\\ \; & +\beta_{6}{\mathrm{lnTExpend}}_{it}+\beta_{7}{\mathrm{MediRatio}}_{it}+\beta_{8}{\mathrm{HospRatio}}_{it}+\beta_{9}{\mathrm{IPGDP}}_{it}\\ \; & +\beta_{10}{\mathrm{lnLPEHealth}}_{it}+{\mathrm{YearDummies}}_{t}+E_{i}+e_{it} \end{array}$$

## 3. Results

### 3.1. Description of Variables

Table 3 shows descriptive statistics and a correlation matrix of the variables. We checked for the multicollinearity problem among variables using variance inflation factor (VIF) before the main test. The average VIF test result value was lower than 5, therefore, the multicollinearity among these variables is not a serious problem.

### 3.2. Impacts of FFS on Hospitals Operations

The detailed results are shown in Table 4:

- Model 1 shows a significant negative relationship between medical revenue and FFS (β=−0.110, p<0.01). Since the dependent variables are taken as natural log, we need to transform back. The result indicates that the annual medical revenue of hospitals with FFS is 89.58% ($e^{-0.11}=0.8958$) of the medical revenue of hospitals without FFS adoptions, which means that public hospitals with FFS make 11.42% less medical revenue.
- In Model 2, the coefficient of FFS (β=−0.329, p<0.01) is negative and statistically significant, indicating that the LOS in public hospitals with FFS reimbursement is 71.96% ($e^{-0.33}=0.7196$) of the LOS of the other public hospitals with no FFS adoption.
- In Model 3, we can also observe a statistically significant association between FFS and lnTMS (β=−0.110, p<0.05), suggesting the medical service capacity of hospitals with FFS is 11.42% ($(1-e^{-0.11})\times 100$% =11.42%) smaller than the others.
- In Model 4, the case of sustainability performance, the FFS coefficient (β=−0.114, p<0.05) suggests that hospitals with FFS invest 10.77% ($(1-e^{-0.114})\times 100\%=10.77\%$) less in personnel training compared to the other hospitals without FFS.

### 3.3. Interaction Impacts of FFS and COH on Hospitals Operations

**Figure 1:**
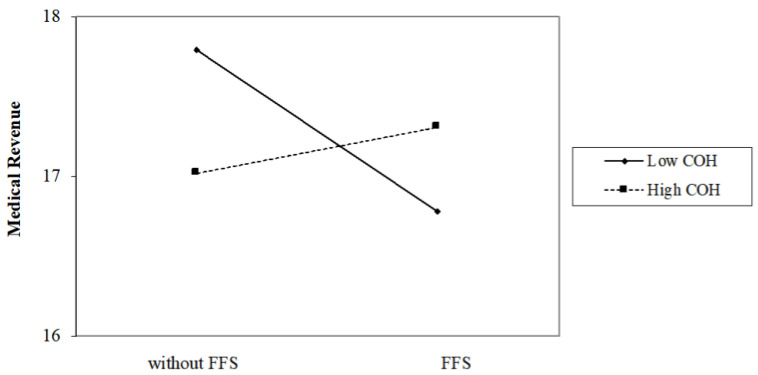
Interaction effect of COH and FFS on Financial income.

**Figure 2:**
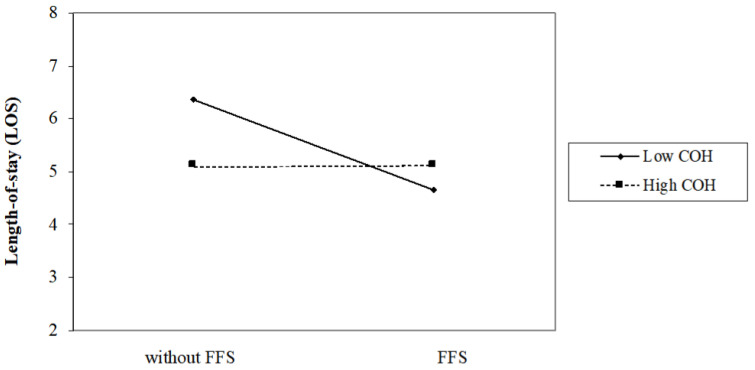
Interaction effect of COH and FFS on efficiency.

**Figure 3:**
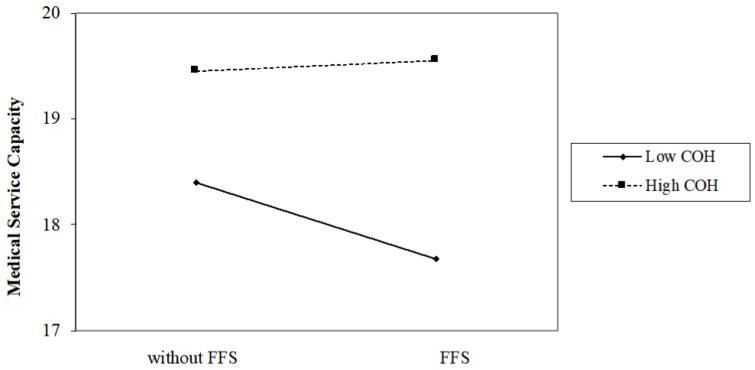
Interaction effect of COH and FFS on medical service capacity.

When considering the interaction between COH and FFS, the outcomes changes. Table 5 presents the interaction effects of COH and FFS on hospitals operations:

- Model 5 of Table 5 shows that in grade II hospitals, the annual medical revenue of hospitals with FFS is 83.36% ($e^{-0.182}=0.8336$) of that hospitals without FFS, while in grade III hospitals, the medical revenue of hospitals with FFS is 115.26% ($(e^{-0.182}\times e^{-0.324})=1.1526$) of that hospitals without FFS. So as shown in Figure 1, FFS has a significant negative impacts on the annual medical revenue on grade II hospitals, but a positive impacts on grade III hospitals.
- As shown in Figure 2, Model 6 states that FFS has a significantly negative effect on LOS on public grade II hospitals ($e^{-0.426}=0.6531$), while it has a significantly positive effect on LOS on public grade III hospitals ($(e^{-0.426}\times e^{-0.431})=1.005$).
- As shown in Figure 3, model 7 indicates that COH significantly weakens the negative effects of FFS on the total number of medical services provided; Specifically, the total number of medical services of hospitals with FFS is 85.64% ($e^{-0.155}=0.8564$) of that hospitals without FFS in grade II hospitals, while in grade III hospitals, the total number of medical services of hospitals with FFS is 104.81% ($e^{-0.155}\times e^{-0.202}=1.0481$) of that hospitals without FFS.
- In Model 8, little evidence shows that the interaction of COH and FFS would affect the amount of investment in personnel training in public hospitals.

### 3.4. Self-Selection Bias Test

We use a random sampling method by pooling data for regression to eliminate the differences in estimated regression coefficients between grade II and grade III hospitals are directly caused by selection bias, We generate the sampling distribution for each interaction coefficient $\beta_{3}$ in Equation (2) using bootstrap for 1000 times with each randomly selected sample size of 301 hospitals (Supplementary Materials). The division between grade II and grade III hospitals remains at 78%/22%. To test the significance of the interaction coefficient, we set the null hypothesis to $\beta_{3}=0$, and the alternative hypothesis should be $\beta_{3}\neq 0$. We calculate a 95% confidence interval for each interaction coefficient by setting the significance level at 0.05 to check if 0 does not fall within the 95% confidence interval. If so, we should reject the null hypothesis [36,37,38,39] and confirm that the estimated differences in FFS impact between grade II and grade III hospitals do not vanish when using a randomized pooled sample. Our results have no self-selection bias. The results are summarized in Table 6.

As we can observe that the 95% confidence intervals for interaction coefficient $\beta_{3}$ of Model 5–8 are [0.3115, 0.3268], [0.4168, 0.4350], [0.1932, 0.2203] and [−0.0126, 0.0309] respectively, where 0 does not fall within 95% confidence interval in Model 5, Model 6 and Model 7, but falls in Model 8. In these cases, we reject the null hypothesis for Model 5, Model 6, and Model 7. We can eliminate the effects of self-selection bias on our regression results by concluding that the estimated difference in the impact of FFS and COH on financial income, efficiency, and medical service capacity does not vanish when using a randomized pooled sample. However, we still cannot observe the impact of FFS and COH on sustainability, where these results are the same as before.

### 3.5. Robustness Test Results

We lagged one year of dependent variables to test the robustness of the results (Table 7), which shows that the estimation results are similar to prior ones. Overall, these estimation results provide empirical evidence regarding our main research question.

## 4. Discussion

### 4.1. Summary and Discussion about Results

At present, the main challenge of China’s medical and health system reform is how to effectively and rationally allocate medical resources, and control the medical costs for patients to meet the basic health service needs. The payment method, known as an effective means to regulate the behaviors of medical services providers, changes the quantity and quality of medical services by influencing the incentives of medical institutions, thus, promoting the rational allocation of medical resources. Since FFS is still the most prevailing payment method in low and middle-income countries (LMICs), analyzing the impacts of FFS on public hospitals’ operations may play an inspiring role for reference to the following health insurance payment reform.

#### 4.1.1. Without Considering COH

Are our results the same as previous research?

- New perspective: The previous research demonstrated that FFS provides incentives for excessive treatment intensity and results in rapid medical costs increase [8,19]. However, most of them took the analysis from a social welfare perspective, while few from hospital operations consider the impacts on hospitals. In contrast, The current study identified significant effects of FFS on public hospitals’ operational outcomes regarding financial income, efficiency, medical service capacity, and sustainability.
- Counterintuitive results: Our empirical results show that FFS negatively affects public hospitals’ medical revenue and medical service capacity, while a positive effect on efficiency by shorter LOS. These results are counterintuitive to common sense that FFS easily leads to higher medical revenue and longer LOS. Based on previous studies [40], patients are more likely to be hospitalized because policies reimburse more than outpatients. Thus, this treatment is preferred for both patients and doctors for financial purposes. Moreover, the number of beds in public hospitals has experienced rapid growth since the new health reform in China, especially for grade III hospitals [14]. The medical capacity is supposed to be increased under FFS, which is inconsistent with our result that the impact of FFS on medical service capacity is negative.
- Why we need to consider COH to explain the counterintuitive results: We observe that the number of grade II hospitals accounts for 78% of our sample. This fact enables us to identify the significant impacts of COH on these four hospitals’ operation outcomes and further analyze whether there is an interaction effect between COH and FFS on hospital operations. This may explain why the results of financial income, efficiency, and medical service capacity are counterintuitive.
- Sustainability: In addition, our study provide the first demonstration that FFS hinders the sustainable development of public hospitals in terms of less investment in personnel clinical training. This result may be since the health care delivery system continues to be hospital-centric under the FFS scheme [5]. The incomes of hospital directors and physicians are still tied to hospital profits [5], namely that health care providers can prescribe a set of stereotypical clinical tests and examinations to achieve performance and earn decent revenues. So without the pressure of financial risk, they have no incentives to make innovative improvements in technology and service processes, which diminishes their motivation to shift from treatment-based curative care to population-based health prevention and management [5,41].

#### 4.1.2. Considering COH

Why grade II hospitals and grade III hospitals perform totally different under FFS?

- Information asymmetry and supply-induced demand under FFS: Our empirical results confirmed that FFS has different effects on hospitals’ operations based on different COH, which is a consequence of the interaction between FFS and COH. Previous research has shown that a principal-agent relationship exists between the supplier and demand side of health care services in the medical market, and there is a strong information asymmetry in this relationship. [42]. However, FFS does not reduce information symmetry to constrain health care providers’ behaviors and may have opposite effects [28]. Specifically, the interests between the principal (patients) and the agent (physicians) are inconsistent. More importantly, the information asymmetry between the two makes it possible for doctors to use their information advantage to influence the needs of patients. Supplier-induced Demand (SID) problems and overprovisions arise when doctors use their information advantages to recommend or provide medical services that may not be necessary for patients’ needs under FFS [43]. Furthermore, these problems are more frequent when medical resources are abundant [18,44].
- COH exacerbates IA and SID under FFS: Medical resources are abundant in China [44]. However, Grade III hospitals in China have suffered from an over-reliance [45] in the past 20 years due to the unbalanced medical resources distribution in China among COH. On the one hand, grade III hospitals attract many patients for their high grade [45] to have a substantial resource siphon capacity. On the other hand, higher grade hospitals can also actively attract highly qualified physicians to serve in high-grade hospitals [17]. Moreover, as we mentioned before, grade II and grade III are also different in handling diseases. Public grade III hospitals can provide more specialized medical services to solve critical and complicated medical conditions. Thus, grade III hospitals have stronger information advantages in complex diseases and are more inclined to induce patient’s needs [14,19], resulting in more treatments in grade III hospitals, more medical revenue, and longer LOS under FFS. Motivated by the social reputation of grade III hospitals, the majority of patients with a solid willingness to avoid health risks are attracted to grade III hospitals [14,40,45]; thus, grade II hospitals would have no competitiveness when offering the same medical services as grade III provide under FFS, which may have a negative effect on grade II hospital’s financial income, shorter LOS and lower medical service capacity under FFS. Therefore, shorter LOS done not implicate grade II hospitals are more efficient under FFS. Alternatively, LOS becomes shorter is raised by more patients are going to grade III hospitals and leads to droping of grade II hospitals’ operational efficiency under FFS.

Why do grade II hospitals with FFS perform differently compared to others with non-FFS?

- FFS vs. single-disease payment (non-FFS): Compared to the grade II hospitals with FFS, the other grade II hospitals adopting payment by single-disease may have a better financial performance result for attracting more patients. Single disease payment is another prevailing payment method in China besides FFS, which refers to the health care providers receiving a lump sum payment for the entire episode of diagnosis and treatment for a single independent disease without complications [46]. The diagnosis selected under such payment is relatively simple with a fixed clinical pathway [47]. So, the treatments are relatively uniform, and the quality of medical care will not be significantly different based on COH. COH affects the pricing of medical services [14], where the payment by single-disease payment for the same diagnosis is lower in grade II hospitals. Because of the commonness of these diseases and the fixation of clinical pathways, the information is relatively symmetrical between doctors and patients, and there are strong alternatives to services among COH. Patients with such diagnoses would choose to go to grade II hospitals for cost-effectiveness. It is difficult for hospitals to induce patients in these diseases and lack the motivation to induce patients. Grade II hospitals can still appeal to patients with cost-effective services paid by single-disease than hospitals in FFS.
- Sustainability: When considering the impacts of COH, there is a lack of evidence showing that the interaction of COH and FFS would affect the amount of investment in personnel training. This may be related to the limited period of our data set; four years of data are insufficient to capture the differences in sustainable development among different levels of hospitals under the FFS scheme.

### 4.2. Strength and Limitations

Our study has some novel findings which could shed some lights on policymakers. To our knowledge, this is the first empirical analysis in China to investigate the impacts of reimbursement schemes on Chinese public hospitals, and to critically compare the differences between different levels of hospitals through the operating outcomes of public hospitals. Our findings are necessary because previous studies on medical operations management have been conducted from the perspective of social welfare, rarely from hospital’s operations, and our research can fill the gap in the related fields. In addition, our study advances the health care operations as well as hospital management literature by empirically testing the impacts on hospitals operations with or without FFS adoption and attempts to give theoretical explanations on the different performances of FFS under COH. Past research mainly studies the hospitals operations either by modelling or only on part of hospitals operations, while our analysis based on previous studies, adds several dimensions to evaluating hospital operations by making full use of grade II and grade III public hospitals operating data. Finally, our study takes the sustainability development trend of Chinese public hospitals into account for the first time. One interesting finding in our analysis shows that FFS reimbursement scheme reduces the enthusiasm of public hospitals to invest in carrying out clinical training; this result broadens the boundaries in studies of health care reimbursement schemes and hospital management.

We acknowledge that there are some limitations in the study design which deserves further research. First, the variables chosen for hospitals operating are from Department of health commission and Department of healthcare security administration at the aggregate level, which only represents a subset of all the operating outcomes at hospitals; so future studies can extend our research by using more granular level data (e.g., patient level). Second, the analysis conducted in this research controls major for hospital level fixed effects; some other factors that may also impact the dependent variables in our study, such as medical operating costs, patients’ responses and caregivers’ satisfaction. Third, the short period of time taken for analysis and limited availability of public hospitals operating data for only one province limits the value of this research, and the future studies might increase sample size and use more diverse hospital data sets (e.g., different classification of hospitals).

### 4.3. Future Work

Managerial contributions

The findings in our research can shed light on both hospital administrators and government regulators:

- First of all, as for hospital administrators, health care providers would have preferred FFS since they can be compensated for delivering the best care to patients based on their professional medical knowledge. Under the background of medical and health system reform, the hospital’s performance goals should be highly consistent with the direction of medical reform. The medical reform emphasizes that all relevant departments should strengthen the application of hospital performance appraisal results. However, our findings can reflect some of the problems existing in grade II and grade III hospitals. Grade II hospitals administrators may consider attracting more patients by improving social reputation and demonstrating their medical ability, such as voluntary consultations and lectures, cooperation with primary health care providers. Besides, the lack of sustainable development under FFS has sounded an alarm for the long-term development of public hospitals. To maintain their disciplinary advantages, public grade II and grade III hospitals should devote themselves to medical research, optimize clinical pathways and carry out clinical personnel training.
- As for health care policymakers, our results confirm that the two types of hospitals perform differently under the same scheme. Grade III hospitals have overwhelmed grade II hospitals regarding medical revenue, utilization of medical resources, and medical service capacity. Thus, the future medical payment reform may consider the systematic variations between these two types of hospitals and reflect those differences in policies, which guide both hospitals’ reform in the same directions.

Theoretical contributions

Our study advances the health care operations and hospital management literature by empirically testing the impacts on hospital performance with or without FFS adoption and considering the interaction impacts of FFS and COH.

- We present a theoretical analysis framework to explore the impacts of payment methods on hospital operation outcomes from financial income, efficiency, medical service capacity, and sustainability. Future analysis of medical insurance payment on hospital performance could use our analytical framework to explore the impacts of other payment methods on hospital’s operation, such as Diagnosis-Related Group (DRG) and Big Data Diagnosis-Intervention Packet (DIP).
- Besides, based on our framework, future research can expand the dimensions to analyze more operation management problems in health care and hospital research fields. Another interesting future research direction could be analyzing how grade II hospitals survive or operate under conditions with low medical revenue in FFS.

## 5. Conclusions

Whether FFS an evil payment scheme that must be stamped out? Our results from the perspective of hospitals operations outcomes indicates that it depends on COH. For public grade II hospitals, nothing is good. The main reason is that it is less attractive to patients; patients are inclined to seek for medical services from higher-level providers [19]. For public grade III hospitals, it is benefit for the financial support and the scale development of hospitals. However, it is detrimental to hospital’s operational efficiency and sustainable development in the long-term. In addition, our findings also confirm that under the interaction impacts of FFS and COH, the problems of information asymmetry and supply-induced demand is more acute in grade III hospitals, and the resources in grade II hospitals are not fully utilized. Thus, the medical resources are still out of balance, and the goal of graded diagnosis is difficult to achieve.

Our analysis could serve as a reference for the health insurance payment reform. There is no perfect payment scheme can fit all grades of hospitals, and our results show that the payment method should have a guiding role in different aspects on different levels of hospitals. To be more specific, For grade III hospitals, the reform of payment mode should guide medical institutions to carry out medical technology innovation and specialize in the treatment of difficult and complicated diseases by improving the high-level development of their medical technology, which indirectly promote the sinking of mild patients, and achieve hierarchical diagnosis and treatment. For public grade II hospitals, by taking advantage of the medical cost differences, the emphasis of health insurance payment reform should be on the improvement of their ability to provide basic medical services, to attract and receive patients, especially those with mild diseases.

## Figures and Tables

**Table 1:** Hospital structural characteristics. Note that the hospital variables were collected in 2019.

		Frequency	Percentage (%)
**Hospital size (Number of beds)**	1000	234	77.74
	1001–2000	45	14.95
	2001–3000	11	3.65
	3001–4000	2	0.66
	4000	9	2.99
**Hospital level**	Grade III	64	21.26
	Grade II	237	78.74
**FFS adoption**	FFS	254	84.39
	Without FFS	47	15.61
	total	301	100
**Grade II hospitals**	FFS	201	84.81
	Without FFS	36	15.19
	total	237	100
**Grade III hospital**	FFS	53	82.81
	Without FFS	11	17.19
	total	64	100

**Table 2:** Key dependent, independent, and control variables: we comprehensively consider the long-term development of the public hospitals from the following perspectives: financial income, efficiency, medical service capacity, and sustainability. Note that the hospital-level variables were collected in 2019.

	Variables Name	Abbreviations	Variable Measurement
**Dependent variables**	Financial income	lnMediRevenue	Natural log of annual medical revenue
	Efficiency	lnLOS	Natural log of average length-of-stay
	Medical Service Capacity	lnTMS	Natural log of outpatients and discharge patients
	Sustainability	lnIPT	Natural log of amount of money invest in personnel training
**Independent variable**	Payment scheme	FFS	Hospital uses FFS as dominant payment FFS = 1, others = 0
**Control variables**	Hospital level	IsGrade	Grade III: IsGeade = 1, Others = 0
	Hospital size	InBeds	Natural log of number of patient beds within a hospital
	Staff number	lnNFE	Natural log of number of full-time employees
	Total expenditure	lnTExpend	Natural log of annual total hospital expenses
	Ratio of medical income	MediRatio	The ratio of medical income from health insurance funds
	Ratio of hospitalization income	HospRatio	The ratio of hospitalization income from health insurance funds
	Economic development	IPGDP	Index of per capita GDP
	Local public expenditure in health	lnLPEHealth	Natural log of local total expenditure in healthcare

**Table 3:** Descriptive statistics and correlation matrix.

Variables	(1)	(2)	(3)	(4)	(5)	(6)	(7)	(8)	(9)	(10)	(11)	(12)	(13)
**(1) lnMediRevenue**	1.00												
**(2) lnLOS**	0.06 *	1.00											
**(3) lnTMS**	0.70 ***	−0.14 ***	1.00										
**(4) lnIPT**	0.60 ***	−0.01	0.52 ***	1.00									
**(5) FFS**	0.09 ***	−0.26 ***	0.06 **	0.05 *	1.00								
**(6) lnBeds**	0.62 ***	0.27 ***	0.42 ***	0.38 ***	0.05 *	1.00							
**(7) lnNFE**	0.94 ***	−0.02	0.70 ***	0.59 ***	0.13 ***	0.61 ***	1.00						
**(8) lnTExpend**	0.55 ***	−0.06 **	0.43 ***	0.64 ***	0.09 ***	0.36 ***	0.53 ***	1.00					
**(9) MediRatio**	−0.12 ***	0.34 ***	−0.18 ***	−0.11 ***	−0.13 ***	0.10 ***	−0.07 ***	−0.06 **	1.00				
**(10) HospRatio**	−0.09 ***	0.24 ***	−0.13 ***	−0.11 ***	−0.10 ***	0.05 *	−0.08 ***	−0.03	0.74 **	1.000			
**(11) IPGDP**	−0.21 ***	−0.02	−0.22 ***	−0.16 ***	−0.01	−0.10 ***	−0.17 ***	−0.13 ***	0.01	−0.01	1.00		
**(12) lnLPEHealth**	0.01	−0.09 ***	0.04	0.13 ***	0.02	0.02	0.00	0.06 **	−0.08 ***	−0.06 **	−0.13 ***	1.00	
**(13) IsGrade**	0.65 ***	0.02	0.60 ***	0.56 ***	−0.02	0.36 ***	0.64 ***	0.42 ***	−0.18 ***	−0.20 ***	−0.20 ***	0.04	1.00
**Mean**	18.4	2.24	11.17	12.38	0.85	6.06	5.89	17.79	0.40	0.49	107.5	3.79	0.21
**SD**	1.45	0.57	1.68	2.54	0.36	1.28	0.96	2.99	0.19	0.19	0.90	0.32	0.40
**Min**	14.3	1.22	4.87	1.47	0	2.63	2.83	6.78	0.01	0.03	105	2.53	0
**Max**	22.52	4.78	15.14	17.94	1	12.54	8.46	22.57	0.99	0.99	109.4	4.27	1

Note that *** indicates p<0.01, ** indicates p<0.05, and * indicates p<0.1.

**Table 4:** Impacts of FFS on medical revenue, LOS, total number of medical services and the amount of investment in personnel training.

Variables	Model 1: lnMediRevenue	Model 2: lnLOS	Model 3: lnTMS	Model 4: lnIPT
**FFS**	−0.110 *** (−8.021)	−0.329 *** (−33.06)	−0.110 ** (−5.078)	−0.114 *** (−3.610)
**IsGrade**	−0.212 *** (13.23)	−0.168 *** (7.715)	0.901 *** (26.41)	1.500 *** (39.32)
**lnBeds**	0.083 *** (10.95)	0.177 *** (98.02)	0.0271 (1.303)	0.0285 (1.284)
**lnNFE**	1.233 *** (57.91)	−0.152 *** (−12.28)	−0.890 *** (20.21)	0.551 *** (22.46)
**lnTExpend**	0.0291 *** (11.67)	−0.0162 *** (−17.63)	0.028 *** (14.45)	0.355 *** (73.44)
**MediRatio**	−0.699 ** (−3.749)	0.814 *** (12.36)	−1.421 *** (−8.226)	0.143 *** (0.262)
**HospRatio**	0.411 * (3.085)	−0.0245 (−0.230)	0.626 ** (3.401)	−0.618 (−1.387)
**IPGDP**	−0.0616 * (−2.925)	−0.0218 (−1.362)	0.125 ** (−4.764)	−0.0115 (−0.137)
**lnLPEHealth**	−0.0674 ** (−4.481)	−0.138 *** (−8.347)	−0.0477 (1.143)	0.726 *** (10.59)
**Constant**	17.10 *** (7.618)	5.48 * (2.855)	18.69 ** (6.126)	1.141 (0.158)
**Year controlled**	Yes	Yes	Yes	Yes
**Observations**	1204	1204	1204	1204
**R-squared**	0.890	0.281	0.556	0.548

Robust t-statistics in parentheses: *** indicates p<0.01, ** indicates p<0.05, and * indicates p<0.1.

**Table 5:** Interaction effect of COH and FFS on focal relationships.

Variables	Model 5: lnMediRevenue	Model 6: lnLOS	Model 7: lnTMS	Model 8: lnIPT
**FFS**	−0.182 *** (−9.586)	−0.426 *** (−43.44)	−0.155 *** (−5.523)	−0.119 *** (−14.73)
**IsGrade**	−0.0591 ** (−3.212)	−0.193 *** (−9.051)	0.732 *** (18.49)	1.480 *** (12.33)
**FFS_isGrade**	0.324 *** (11.01)	0.431 *** (66.00)	0.202 ***(5.948)	0.0233 (0.162)
**lnBeds**	0.079 *** (10.68)	0.172 *** (96.11)	0.0245 (1.183)	0.0282 (1.194)
**lnNFE**	1.237 *** (58.87)	−0.148 *** (−11.71)	0.892 *** (20.31)	0.552 *** (21.40)
**lnTExpend**	0.0290 *** (12.19)	−0.0163 *** (−18.05)	0.0279 *** (14.83)	0.355 *** (73.59)
**MediRatio**	−0.714 ** (−3.791)	0.795 *** (12.32)	−1.431 *** (−8.207)	0.142 (0.263)
**HospRatio**	0.424 * (3.144)	−0.0070 (−0.068)	0.634 ** (3.416)	−0.617 (−1.401)
**IPGDP**	−0.0615 ** (−3.358)	−0.0218 (−1.765)	−0.125 ** (−4.599)	−0.0115 (−0.173)
**lnLPEHealth**	−0.0818 *** (−6.472)	−0.157 *** (−10.20)	0.0387 (0.895)	0.725 *** (9.766)
**Constant**	17.22 *** (8.897)	5.310 ** (3.791)	18.77 *** (5.938)	1.149 (0.159)
**Year controlled**	Yes	Yes	Yes	Yes
**Observations**	1204	1204	1204	1204
**R-squared**	0.891	0.293	0.556	0.548

Robust t-statistics in parentheses: *** indicates p<0.01, ** indicates p<0.05, and * indicates p<0.1.

**Table 6:** Self-selection bias test results using bootstrap.

		lnMediRevenue	lnLOS	lnTMS	lnIPT
**Number of Bootstrap**		1000			
**A random sample size each time**		301 (237 grade II and 64 grade III)			
**Number of *p*-value < 0.05**		978	988	787	421
**Mean** ($\beta_{3}$)		0.3192	0.4259	0.2608	0.0092
$\beta_{3}$ **in Table 5**		0.324	0.431	0.202	0.0233
**95% CI**	lower	0.3115	0.4168	0.1932	0.0309
	upper	0.3268	0.435	0.2203	0.0309

**Table 7:** Robustness test results of lagged dependent variables.

Variables	Model 9: lnMediRevenue_lag	Model 10: lnLOS_lag	Model 11: lnTMS_lag	Model 12: lnIPT_lag
**FFS**	−0.168 *** (−5.184)	−0.416 *** (−74.22)	−0.167 * (−4.071)	−0.101 ** (−5.097)
**IsGrade**	−0.0231 (−0.836)	−0.161 ** (−7.853)	0.748 *** (19.10)	1.541 *** (14.67)
**FFS_isGrade**	0.292 ** (7.785)	0.417 *** (30.42)	0.153 * (3.722)	−0.0452 (0.0255)
**lnBeds**	0.0706 *** (10.73)	0.174 *** (64.27)	0.0267 (1.489)	0.0255 (1.263)
**lnNFE**	1.248 *** (92.17)	−0.155 *** (−20.60)	0.914 *** (36.70)	0.544 *** (25.59)
**lnTExpend**	0.0237 ** (9.185)	−0.0162 *** (−9.966)	0.0234 *** (9.965)	0.357 *** (82.15)
**MediRatio**	−0.468 (−2.435)	0.768 ** (6.874)	−1.268 *** (−17.20)	0.676 (1.547)
**HospRatio**	0.276 (1.805)	0.0496 (0.446)	0.446 ** (5.256)	−1.195 * (−3.619)
**IPGDP**	−0.0646 * (−2.990)	−0.0241 (−1.589)	−0.109 ** (−7.817)	−0.00736 (−0.0881)
**lnLPEHealth**	−0.0711 * (−4.207)	−0.172 *** (−10.98)	0.0537 (1.503)	0.740 *** (21.10)
**Constant**	17.47 ** (7.459)	5.619 * (3.302)	16.89 ** (9.832)	0.609 (0.0677)
**Year controlled**	Yes	Yes	Yes	Yes
**Observations**	903	903	903	903
**R-squared**	0.892	0.301	0.561	0.548

Robust t-statistics in parentheses: *** indicates p<0.01, ** indicates p<0.05, and * indicates p<0.1.

## Supplementary Materials

The following are available online at https://www.mdpi.com/article/10.3390/ijerph182312723/s1.

## Abbreviations

The following abbreviations are used in this manuscript:

FFS	Fee-for-Service
LMICs	Low and middle-income countries
PPS	Prospective payment system
BP	Bundle payment
VBP	Value-based purchasing
HIT	Health information technology
DID	Difference-in-Differences
LOS	Length-of-stay
MSC	Medical service capacity
NHC	National health commission
VIF	Variance inflation factor
IA	Information asymmetry
SID	Supply-induced demand
DRG	Diagnosis-Related Group
DIP	Big Data Diagnosis-Intervention Packet
